# Desmoid-type fibromatosis in an uncommon location: A case report of shoulder involvement misdiagnosed as rhabdomyosarcoma

**DOI:** 10.1016/j.ijscr.2024.110508

**Published:** 2024-10-23

**Authors:** Faten Limaiem, Mohamed Amine Gharbi, Nadia Boujelbene, Ramy Triki, Khaled Ben Romdhane, Ramzi Bouzidi

**Affiliations:** a University of Tunis El Manar, Tunis Faculty of Medicine, 1007, Tunisia; b Pathology Department, Hospital Mongi Slim La Marsa, Tunisia; c Department of Orthopedic Surgery, Hospital Mongi Slim La Marsa, Tunisia; d Salah Azaïez Institute, Tunis, Tunisia; e Privatee Pathology Laboratory, Tunisia

**Keywords:** Musculoskeletal neoplasms, Desmoid tumor, Fibromatosis, Shoulder, Surgery, Pathology

## Abstract

**Introduction and importance:**

Desmoid-type fibromatosis is an uncommon tumor characterized by its local invasiveness, with shoulder involvement being notably infrequent. The optimal treatment strategy for this tumor remains a topic of ongoing debate.

**Case presentation:**

A 47-year-old Tunisian woman with a history of hypothyroidism, presented with pain and swelling in her left shoulder for a year. Examination revealed a firm, painful 4 cm mass, and MRI showed a poorly defined intramuscular tumor in the deltoid muscle, initially suspected to be rhabdomyosarcoma. However, surgical biopsy confirmed desmoid-type fibromatosis of the shoulder. The patient underwent surgical wide en-bloc resection of the tumor. The patient's recovery was uneventful, and she received physical therapy. At the three-year follow-up, she reported residual shoulder pain after heavy lifting, improving with analgesics. Examination showed no neurological deficits and a Constant score of 83 out of 100.

**Clinical discussion:**

Due to their deep-seated nature and infiltrative growth patterns into neighboring subcutaneous tissues or muscles, along with the presence of myxoid or fibrotic components, desmoid-type fibromatosis can present challenges in distinguishing them from malignant soft tissue neoplasms based on imaging characteristics.

**Conclusions:**

While radiologic evaluations may indicate characteristics suggestive of a malignant soft tissue tumor, histological confirmation is imperative prior to initiating surgical intervention. Continued research into the optimal treatment approaches for desmoid-type fibromatosis is essential for improving future patient outcomes and quality of life.

## Highlights

• Desmoid-type fibromatosis is a rare, locally aggressive tumor that seldom metastasizes and is uncommon in the shoulder.
• Desmoid-type fibromatosis presents a diagnostic challenge due to its rarity and varied clinical manifestations.
• MRI is the favored imaging technique, and an imaging-guided core-needle biopsy is crucial for establishing a correct diagnosis.
• Histological confirmation is essential before beginning treatment, even if clinical and radiological assessments suggest malignancy.
• Desmoid tumors lack a standard treatment, with 20% experiencing spontaneous regression, leading to ongoing debate on the optimal therapy.

## 1 Introduction

Desmoid-type fibromatosis, as defined by the World Health Organization, is a fibroblastic proliferation characterized by infiltrative growth, local recurrence, and non-metastatic behavior in deep soft tissues [1,2]. Although classified as benign, these tumors are known for their tendency to recur locally after treatment. Desmoid tumors can arise in various regions of the body, with the abdominal wall and intra-abdominal cavity being the most frequent sites of occurrence [2]. However, shoulder involvement is particularly rare, resulting in a limited understanding of its clinical presentation, diagnostic challenges, and management strategies. Specifically, the literature lacks comprehensive descriptions of symptoms, imaging findings, and effective treatment approaches for desmoid-type fibromatosis in this location. This case report presents a rare instance of desmoid-type fibromatosis in the shoulder of a 47-year-old woman. By detailing the diagnostic approach, treatment plan, and postoperative outcomes, the authors aim to enrich the current knowledge regarding shoulder fibromatosis and review the relevant literature.

This case report adheres to the SCARE Criteria [3].

## 2 Case presentation

**Fig. 1:**
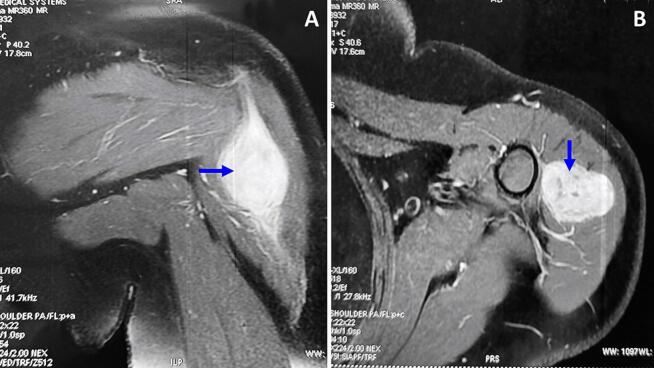
A: Coronal MRI showing the intramuscular tumor within the left deltoid muscle (Blue arrow). B: Axial MRI displaying the lobulated and indistinct lesion (Blue arrow) with a heterogeneous signal, appearing hyperintense on T2-weighted diffusion images. (For interpretation of the references to colour in this figure legend, the reader is referred to the web version of this article.)

**Fig. 2:**
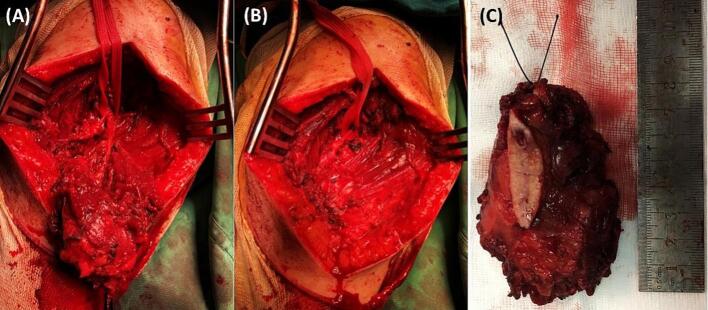
A: showing en-bloc resection of the tumor and its close connection with the axillary circumflex neurovascular pedicle. B: showing the tumor bed after wide resection with intact axillary circumflex neurovascular pedicle. C: showing the resected specimen.

**Fig. 3:**
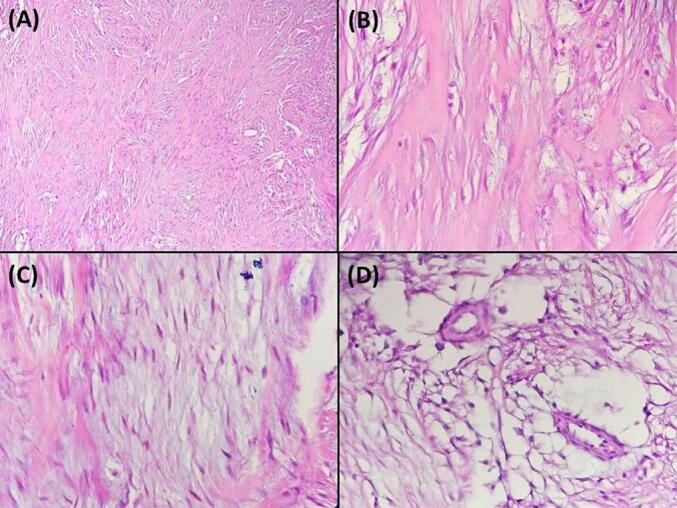
A: Histopathological section illustrating spindle cell proliferation without significant atypia or pleomorphism. This proliferation showcases elongated, slender spindle cells forming extensive sweeping fascicles, characterized by a uniform appearance and pale cytoplasm. **(**Hematoxylin and eosin, magnification × 200**)**. B: Keloid-like regions were observed in specific focal areas. (Hematoxylin and eosin, magnification × 400**)**. C: Focal areas of myxoid alteration were identified **(**Hematoxylin and eosin, magnification × 400**)**. D: Multiple perivascular halos were observed. **(**Hematoxylin and eosin, magnification × 400**)**.

**Fig. 4:**
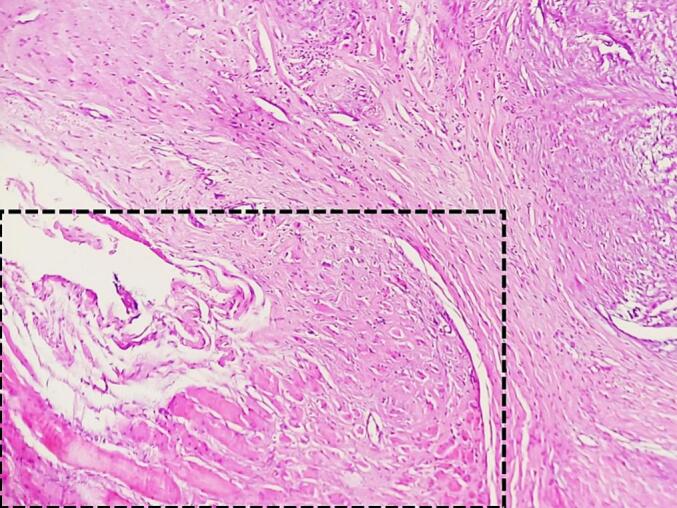
Presence of infiltration into adjacent muscle and soft tissue at the border. The area is framed with hatching. **(**Hematoxylin and eosin, magnification × 200**)**.

**Fig. 5:**
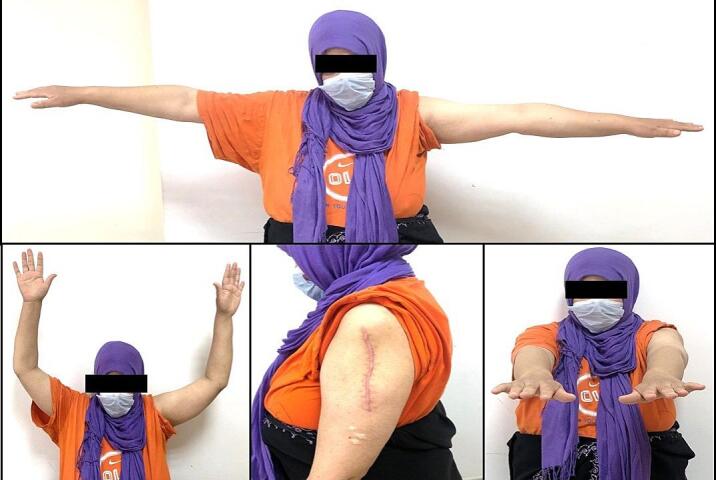
Photograph demonstrating the range of motion in the affected shoulder of the patient.

A 47-year-old Tunisian woman with a medical history of hypothyroidism, managed with Levothyrox 125 μg since 2018, and a surgical history of ventral hernia repair in 2010, presented with a one-year history of pain and swelling in her left shoulder, which had not improved despite medical analgesic treatment. Her family medical history did not reveal any significant conditions. On physical examination, a firm, rounded mass measuring 4 cm in diameter was palpated in the proximal third of the lateral aspect of the left arm. The mass was painful on palpation, fixed relative to surrounding structures, but without signs of local inflammation. Shoulder range of motion was reduced with limited active abduction and anteversion. Constant score was 64/100. There was no neurological deficit and no palpable lymphadenopathy was detected. Magnetic resonance imaging of the left shoulder identified an intramuscular tumor in the left deltoid muscle, measuring 33 × 26 mm with a cranio-caudal extension of 41 mm. The lesion appeared lobulated and poorly defined, displaying a heterogeneous signal that was hyperintense on T2-weighted images and isointense on T1-weighted images (Fig. 1A, B). After contrast administration, intense and heterogeneous enhancement was observed (Fig. 1A, B), along with edema and infiltration of adjacent smooth muscle fibers, which also showed post-contrast enhancement. The MRI suggested a diagnosis of rhabdomyosarcoma. A surgical biopsy of the tumor was performed, and histopathological analysis confirmed the diagnosis of desmoid-type fibromatosis. Considering the patient's young age, the presence of a large resectable tumor involving the deltoid muscle, the persistent disabling shoulder pain that had not improved with symptomatic medical treatment, and the significant functional impairment, a decision was made to proceed with surgical intervention. The patient subsequently underwent surgical wide en-bloc resection of the tumor (Fig. 2A) with careful dissection of axillary circumflex neurovascular pedicle (Fig. 2B), and the resected specimen was sent for pathological evaluation. The specimen measured 7.5 × 5 × 4 cm and was covered with skin, with the excision extending deeply into the fascia (Fig. 2C). Sectioning revealed a 40 mm intramuscular nodule, closely adherent to the fascia, displaying a homogeneous, finely fasciculated, whitish appearance. Histological analysis showed a mesenchymal proliferation of regular spindle cells with elongated nuclei (Fig. 3A), forming long fascicles within a collagenous stroma that was abundant and edematous, featuring dense, hyaline, pseudo-keloid fibrous bands (Fig. 3B). Focal myxoid alterations were present (Fig. 3C), along with a distinct peri-vascular halo (Fig. 3D). The tumor infiltrated adjacent muscle (Fig. 4) and adipose tissue, with lymphoid clusters at the margins. The surgical margins were free of tumor cells, with clear margins exceeding 2 mm. Postoperative recovery was uneventful, and the patient was prescribed physical therapy. At the three-year follow-up, the patient reported residual pain in her left shoulder, particularly after heavy lifting, which improved with analgesics. The physical examination revealed no neurological deficits, and she exhibited a complete range of motion (Fig. 5), with a Constant score of 83 out of 100. No recurrence was observed.

## 3 Discussion

Desmoid-type fibromatosis is a rare condition with an incidence of two to six new cases per million annually, primarily affecting females between puberty and 40 years old [4,5]. It is associated with mutations in the β-catenin gene for sporadic cases and the APC gene for familial cases, often linked to familial adenomatous polyposis syndrome. Sporadic cases generally exhibit localized aggressiveness without metastasis, commonly in the limbs, girdle, trunk, or neck, while familial cases typically occur in the abdomen, affecting the mesentery or intestinal wall [[6], [7], [8]]. Initially, patients may be asymptomatic, with symptoms arising from pain and deformity due to the compression of nearby organs or neurovascular structures, as well as damage to adjacent musculoskeletal tissues [5,7,8]. MRI represents the most reliable diagnostic modality for identifying desmoid-type fibromatosis. Desmoid tumors commonly exhibit a heterogeneous MR imaging pattern, appearing iso- to hyperintense on T2-weighted images and isointense on T1-weighted images compared to skeletal muscle. Signal variations are attributed to collagen density, cellularity, and spindle cell content. Additionally, these tumors typically show moderate to intense contrast enhancement in about 90 % of cases. [9,10]. Due to their deep-seated nature and infiltrative growth patterns, desmoid-type fibromatosis can be difficult to differentiate from malignant soft tissue neoplasms, including sarcomas and melanoma metastases. Malignant soft tissue tumors display heterogeneous signals on T1-weighted and T2-weighted images, with T2 signal intensity greater than that of fat, and are often associated with calcifications or cystic necrosis. The conclusive diagnosis is made through histopathological examination, revealing a proliferation of uniform spindle cells resembling myofibroblasts in abundant collagenous stroma and a vascular network. Immunohistochemical staining shows positive results for nuclear beta-catenin, vimentin, cyclooxygenase-2, platelet-derived growth factor receptor beta, androgen receptor, and estrogen receptor beta, while being negative for desmin, S-100, h-caldesmon, CD34, and c-KIT [9]. Various morphological patterns include conventional, hyalinized, staghorn vessel, myxoid, keloidal, nodular fasciitis-like, and hypercellular patterns [11]. In our case, MRI findings initially suggested rhabdomyosarcoma, but the preoperative shoulder mass biopsy confirmed desmoid-type fibromatosis. The histological differential diagnoses of desmoid-type fibromatosis include scar tissue, which typically exhibits a noninfiltrative growth pattern and is associated with a history of trauma or surgery at the site. Gardner fibromas, on the other hand, are smaller lesions with dense collagen bundles and are commonly linked with Gardner syndrome. Additionally, nodular fasciitis, proliferative fasciitis/myositis, myofibroma/myofibromatosis, calcifying fibrous pseudotumor, and other entities with distinct histological features should be considered. Currently, there is no standardized treatment for desmoid tumors, which can spontaneously regress in up to 20 % of cases [4,5,8]. Surgical resection is increasingly avoided due to high morbidity and recurrence rates, with local recurrences ranging from 15 % to 77 %, averaging 14.1 months [8]. Up to two-thirds of removed lesions may recur, regardless of surgical margins, due to their infiltrative nature affecting surrounding structures [8,12]. In our case, at the three-year follow-up, the patient reported residual shoulder pain after heavy lifting, improving with analgesics. Examination showed no neurological deficits and a Constant score of 83 out of 100. Current medical treatments include anthracycline and vinca-alkaloid chemotherapy, along with targeted therapies such as tyrosine kinase inhibitors [5]. Promising new treatments, including gamma-secretase inhibitors, are awaiting FDA approval [5,8]. Cryoablation is also being explored, and several agents targeting the Wnt pathway are in development [5]. Given the limited efficacy of these options and the potential for spontaneous regression, there is a growing trend toward conservative management with regular monitoring. The Desmoid Tumor Working Group's 2018 consensus emphasized that active surveillance should be the first-line approach for managing desmoid tumors [13]. Guidelines suggest addressing any disease progression primarily with medical therapy, except in cases involving the abdominal wall, where surgery is indicated. This study identified that lesions in the shoulder girdle tend to progress more under active surveillance [13]. Previous research, including Bonvalot et al., indicated that lesions in the limbs are at higher risk compared to those in the trunk and neck [14]. Other authors' findings further confirmed the shoulder girdle as a significant area for desmoid-type fibromatosis progression [8]. Therefore, lesions in this region should be actively treated or closely monitored with shorter follow-up intervals to minimize the risk of significant growth. In conclusion, this case highlights the difficulties in diagnosing desmoid-type fibromatosis and emphasizes the necessity of confirming histology prior to surgical intervention. The successful treatment of a 47-year-old woman with shoulder desmoid-type fibromatosis underscores the importance of accurate diagnosis and timely action. At the three-year follow-up, she reported residual shoulder pain after heavy lifting, which improved with analgesics. Examination revealed no neurological deficits, and she achieved a Constant score of 83 out of 100, reflecting positive outcomes with personalized management. Although the treatment results were promising, the limitations of this report include its focus on a single case and a relatively short follow-up period. Further research into optimal therapies for desmoid-type fibromatosis is essential for improving patient outcomes and quality of life.

## Author contribution

Dr. Faten Limaiem and Dr Nadia Boujelbene: Prepared, organized, wrote, and edited all aspects of the manuscript.

Dr. Mohamed Amine Gharbi, Dr Ramy Triki and Pr. Ramzi Bouzidi: Read, edited, and approved the final version of the manuscript. Contributed to data acquisition, analysis, and interpretation. Provided final approval of the manuscript before its submission.

Pr Khaled Ben Romdhane: Performed the gross and microscopic pathologic evaluation of the pathology specimen.

## Consent

Written informed consent was obtained from the patient for publication of this case report and accompanying images. A copy of the written consent is available for review by the Editor-in-Chief of this journal on request.

## Ethical approval

Ethical approval for this study was provided by the Ethical Committee of Mongi Slim University Hospital, Marsa, Tunisia.

## Sources of funding

This research did not receive any specific grant from funding agencies in the public, commercial, or not-for-profit sectors.

## Guarantor

Dr Faten Limaiem

## Declaration of competing interest

None declared.
